# HD-PTP Is a Catalytically Inactive Tyrosine Phosphatase Due to a Conserved Divergence in Its Phosphatase Domain

**DOI:** 10.1371/journal.pone.0005105

**Published:** 2009-04-02

**Authors:** Marie-Claude Gingras, Yu Ling Zhang, Dmitri Kharitidi, Alastair J. Barr, Stefan Knapp, Michel L. Tremblay, Arnim Pause

**Affiliations:** 1 Goodman Cancer Centre and Department of Biochemistry, McGill University, Montréal, Québec, Canada; 2 Structural Genomics Consortium, Nuffield Department of Medicine, University of Oxford, Oxford, United Kingdom; University of Oldenburg, Germany

## Abstract

**Background:**

The HD-PTP protein has been described as a tumor suppressor candidate and based on its amino acid sequence, categorized as a classical non-transmembrane protein tyrosine phosphatase (PTP). To date, no HD-PTP phosphorylated substrate has been identified and controversial results concerning its catalytic activity have been recently reported.

**Methodology and Results:**

Here we report a rigorous enzymatic analysis demonstrating that the HD-PTP protein does not harbor tyrosine phosphatase or lipid phosphatase activity using the highly sensitive DiFMUP substrate and a panel of different phosphatidylinositol phosphates. We found that HD-PTP tyrosine phosphatase inactivity is caused by an evolutionary conserved amino acid divergence of a key residue located in the HD-PTP phosphatase domain since its back mutation is sufficient to restore the HD-PTP tyrosine phosphatase activity. Moreover, in agreement with a tumor suppressor activity, HD-PTP expression leads to colony growth reduction in human cancer cell lines, independently of its catalytic PTP activity status.

**Conclusion:**

In summary, we demonstrate that HD-PTP is a catalytically inactive protein tyrosine phosphatase. As such, we identify one residue involved in its inactivation and show that its colony growth reduction activity is independent of its PTP activity status in human cancer cell lines.

## Introduction

The classical protein tyrosine phosphatase (PTP) family characterized by a ∼280 amino acid domain containing 10 conserved motifs, is divided into transmembrane receptor-like and intracellular nonreceptor PTPs, [1], [2]. PTP motif 9 (VHCSXGXGR[T/S]G) is one of the most conserved sequences and corresponds to the active site of the enzyme. The cysteine residue (C) present in this motif is essential for catalytic activity and its replacement by a serine residue (S) abrogates the activity (C/S mutant) [1]–[3].

The HD-PTP protein has been described as a tumor suppressor candidate since it is encoded by the *PTPN23* gene, located on the 3p21.3 tumor suppressor gene cluster frequently deleted in human kidney, lung, breast and cervical tumors [4]–[8]. In agreement with a tumor suppressor function, HD-PTP expression inhibits *ras*-mediated transformation of NIH-3T3 cells; this effect is abrogated by deletion of its PTP domain as well as by incorporation of a C/S mutation suggesting that HD-PTP catalytic activity regulates this function [4]. HD-PTP functions in cell migration and endosomal trafficking were also recently reported but its exact cellular activities have not been characterized yet [9]–[12]. Based on its amino acid sequence, HD-PTP has been classified as a non-transmembrane PTP [1], [2], [8]. Importantly, it possesses a 291 amino acids PTP domain which is comprised of 10 PTP motifs that define this enzyme family. While bacterially expressed HD-PTP has recently been reported as catalytically inert, mammalian expressed HD-PTP tyrosine phosphatase activity has been shown by another group [11], [13].

Here we report a detailed and rigorous enzymatic analysis clearly demonstrating that mammalian expressed HD-PTP catalytic domain and full-length proteins do not harbor any PTP activity towards DiFMUP or phosphatidylinositol phosphates *in vitro*. This lack of activity is caused by an evolutionary conserved sequence divergence of a key non-consensus residue located in its PTP motif 9, since its back mutation specifically reactivates HD-PTP tyrosine phosphatase activity against DiFMUP. We also report that the HD-PTP tyrosine phosphatase activity status does not affect its colony growth reduction activity in human cancer cell lines.

## Materials and Methods

### DNA constructs, cloning and site-directed mutagenesis

Human HD-PTP cDNA was kindly provided by Dr. Mamoru Ouchida (Okayama University, Okayama, Japan) [8]. The cDNA encoding the full-length protein (human amino acid 1 to 1636) or the HD-PTP catalytic domain (human amino acids 1169 to 1460) were amplified by PCR using primers containing an EcoR1 site at the 5′ end and an Xho1 site at the 3′ end and were cloned into a pcDNA3 vector in frame with two Flag epitopes using EcoR1 and Xho1 enzymes (2XFlag pcDNA3 vector was previously described [14]). The integrity and the correct insertion of the cDNA were verified by sequencing. The C1392S, the S1394A and the C1392S/S1394A mutants were generated by site-directed mutagenesis with the Quick Change Site-Directed Mutagenesis kit (Stratagene, La Jolla, CA) according to the manufacturer's directions using Flag-HD-PTP full-length and catalytic domain constructs as templates and modified forward and reverse primers described in figure S1. The mutations were confirmed by DNA sequencing. The GST-PTP1B catalytic domain and the GST-PTEN wild type and C/S constructs have been previously described [15], [16].

### Protein expression and purification

HEK 293T cells were maintained in Dulbecco's modified Eagle media (DMEM high glucose) supplemented with 10% fetal bovine serum at 37°C in a 5% CO_2_ humidified atmosphere. Cells were transfected with HD-PTP constructs or an empty vector using Lipofectamine 2000 reagent (Invitrogen, Carlsbad, CA) according to the manufacturer's instructions. Expressed proteins (HD-PTP full-length or catalytic domain) were purified using a Flag purification system (EZview Red ANTI-FLAG M2 Affinity Gel, Sigma-Aldrich, St-Louis, MO): Twenty-four hours post-transfection, cells were harvested, rinsed with phosphate-buffered saline (PBS) and lysed 15 min on ice with HNMETG buffer (50 mM Hepes, 150 mM NaCl, 1.5 mM MgCl_2_, 1 mM EGTA, 10% glycerol, 1% Triton X-100, 5 mM DTT and 1× of the ROCHE protease inhibitor cocktail). Cell lysates were cleared by centrifugation at 13000×g and incubated with Flag beads for 2 hours at 4°C. Beads were washed 3 times with HNMETG containing only 0.1% Triton X-100 and the Flag-HD-PTP protein was eluted 3 times with 150 ng/ml of Flag peptides in phosphate-buffered saline solution (PBS). Eluted proteins were concentrated using Microcon centrifugal Filter Devices (Millipore, Bedford, MA) and quantified using the RC/DC protein assay kit (Bio-Rad, Hercules, CA). Equal amounts of proteins were loaded on SDS-PAGE gel and the presence and the purity of the eluted proteins were evaluated by Coomassie staining and western blot analysis using anti-Flag M2 antibody (Sigma-Aldrich, St-Louis, MO). The GST-tagged catalytic domain of PTP1B and GST-PTEN full-length were purified as described and their expression was evaluated by Coomassie staining and western blot analysis using anti-GST antibody, kindly provided by Dr. N. Sonenberg [15], [16].

### Enzymatic Assays

Tyrosine phosphatase assays were performed at 37° in black polystyrol 96-well plate using the EnzCheck Phosphatase Assay Kit (Molecular Probes, Carlsbad, CA) according to the manufacturer's instructions. Briefly, DiFMUP substrate (100 µM final concentration) was added to assay buffer (50 mM Hepes, 0.1 mg/ml BSA, 3 mM DTT, pH 6.5) containing 100 to 200 nM of Flag-HD-PTP proteins or 2 nM of purified GST-PTP1B in a final volume of 100 µl. The fluorescence emitted at 450 nm by the hydrolyzed DiFMU fluorogenic product was monitored every minute for 30 minutes using the Thermo VARIOSKAN fluorescence plate reader (excitation at 358 nm and emission at 450 nm) and was compared to a standard curve. The rate of the reaction was obtained by dividing the fluorigenic DiFMU produced (pmole) per time (minutes). When indicated, 10 mM of the PTP inhibitor sodium orthovanadate (Fisher Scientific, Ottawa, ON) was added to the buffer [17]. For pH profiling studies, enzymatic reactions were performed at the indicated pH (ranging from 4 to 8.5) in buffer containing 50 mM Tris, 50 mM Bis-Tris and 100 mM sodium acetate to maintain a constant ionic strength as previously suggested [18]. For kinetic analysis, a constant concentration of Flag-HD-PTP was added to different concentration of DiFMUP substrate (final concentration between 0 and 300 µM). The reaction rates were plotted against the DiFMUP concentration to obtain the Michaelis-Menten rectangular hyperbola and kinetic constants (*K_M_, k_cat_,* and *k_cat_*/*K_M_*) were derived from this hyperbola using GraphPad Prism software and compared to known DiFMUP enzymatic parameters of PTP1B, SHP2 and TC-PTP tyrosine phosphatases [19] [20]. The lipid phosphatase assays were performed using the Malachite Green Phosphatase Assay Kit (Echelon) according to the manufacturer's directives. Briefly, the assay was performed by incubating 1 µg of HD-PTP catalytic domain in assay buffer (25 mM Tris-HCl pH 7.4, 140 mM NaCl, 2.7 mM KCl, 10 mM DTT) for 15 min at 37°C with 120 µM of phosphatidylinositol phosphate substrate in a total volume of 25 µl. PTEN (250 ng) was used as a positive control. The reaction was terminated by adding 100 µl of Malachite Green solution, and incubated 15 min at room temperature to allow color development. The absorbance was read at 620 nm using the Thermo VARIOSKAN fluorescence plate reader.

### Colony formation assays

Renal carcinoma cell lines ACHN and 786-0 were maintained as described for HEK 293T cells and were transfected with an empty pcDNA3 vector containing a neomycin resistance gene or with the Flag-HD-PTP constructs as described above. Twenty-four hours after transfection, cells were trypsinized, plated in 60 mm dishes and selected for 2 weeks using G418. The colonies obtained were fixed, stained with Giemsa and counted as described [21]. An aliquot of cells was preserved for protein expression levels by western blot analysis using anti-Flag (M2) antibody and anti-actin (AC74, Sigma-Aldrich, St-Louis, MO) was used as loading control.

## Results

### HD-PTP tyrosine phosphatase activity

**Figure 1:**
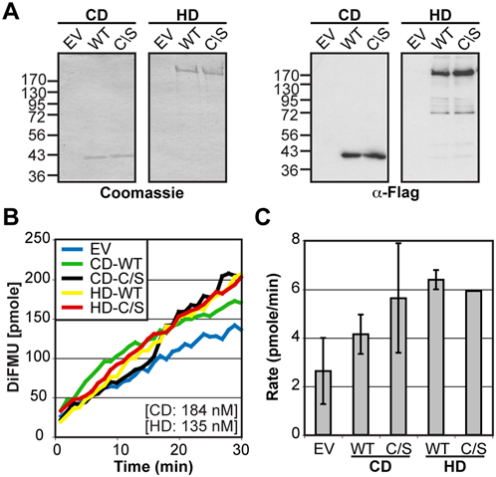
HD-PTP is catalytically inactive. A) Flag-HD-PTP catalytic domain (CD) or full-length protein (HD) wild type (WT) or C1392S (C/S) or the corresponding empty vector (EV) were expressed in HEK 293T cells, purified and visualized by Coomassie staining and western blot analysis using anti-Flag antibody. B) HD-PTP phosphatase activity was performed using DiFMUP substrate and representative time-course kinetic curves of hydrolyzed DiFMU fluorescence emission is shown. C) The average speed of the reactions (Rate) was derived from the kinetic curves and is expressed as pmole of DiFMU per minute (pmole/min). Results represent the average of three independent experiments (+/−SD).

Recombinant full-length PTPs as well as isolated PTP domains exhibit tyrosine phosphatase activity *in vitro* against synthetic substrates and are used to characterize PTP activity [19], [20], 22. However, since PTP activity could be regulated by post-translational modifications that are not present in protein expressed in bacteria, results depend on the expression system used [23]. To clarify the conflicting results regarding HD-PTP catalytic activity, we evaluated the phosphatase activity of the mammalian expressed wild type versions of either Flag-HD-PTP full-length (HD: human amino acid 1 to 1636) or catalytic domain (CD: human amino acids 1169–1460) and compared it to the potential catalytically inactive mutant versions (C1392S: C/S) (Fig. 1). The proteins were expressed in HEK 293T cells, purified and their presence and purity confirmed by Coomassie staining and western blot analysis using anti-Flag antibody (Fig. 1A). The HD-PTP enzymatic activity was assayed using the highly sensitive non-selective fluorogenic substrate 6, 8-Difluoro-4-Methylumbiliferyl Phosphate (DiFMUP) as previously described [19], [20]. The fluorescence emitted by the dephosphorylation of the substrate was monitored every minute for 30 minutes (Fig. 1B) and the rate of the reaction (pmole of DiFMU/min) was derived (Fig. 1C). Importantly, even though a slight difference between the reaction rates of the empty vector (EV) and of the wild type constructs (CD-WT and HD-WT) was observed, the catalytically inactive mutants (CD-C/S and HD-C/S) displayed a background activity similar to the WT. This suggests that HD-PTP is catalytically inactive and that the weak activity detected with these constructs is due to the presence of small amounts of PTPs that co-purified with HD-PTP. These data are in agreement with the absence of activity reported recently using bacterially expressed HD-PTP with a panel of 38 phospho-peptides and the DiFMUP substrate [13].

**Figure 2:**
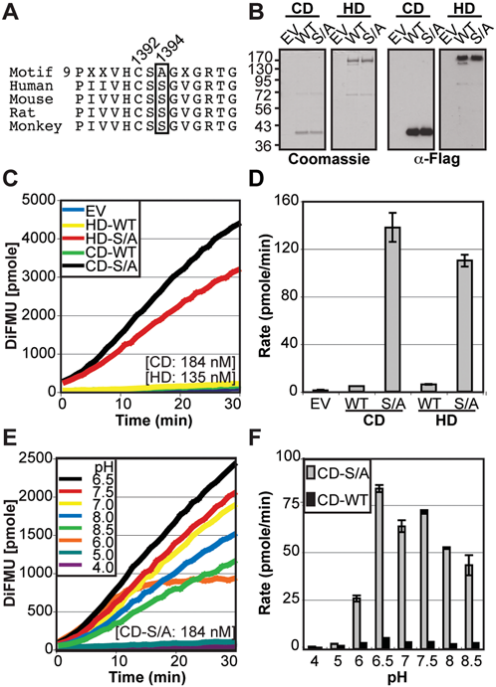
S1394A mutation restores the HD-PTP catalytic activity. A) Alignment of the PTP motif 9 consensus with HD-PTP sequences shows an amino acid divergence conserved among species (human amino acid 1394 is a serine instead of an alanine; boxed). The essential catalytic cysteine residue (C) is indicated. B) Purified catalytic domain of HD-PTP (CD) or full-length protein (HD), wild type (WT) or S1394A (S/A) were visualized by Coomassie staining and western blot using anti-Flag antibody. C–F) Phosphatase assays were performed at pH 6.5 (C, D) or at pH values ranging from 4 to 8.5 (D, E) as described in figure 1. C, E) Representative time-course kinetic curves of hydrolyzed DiFMU fluorescence emission. D, F) Average speed of the reaction (Rate) was derived from the kinetic curves and results represent the average of three independent experiments (+/−SD).

Alignment of the catalytic domain of HD-PTP with the other classical PTP sequences highlighted the presence of many divergences in critically conserved residues [1]. While HD-PTP harbors the cysteine (C1392 in the human HD-PTP sequence) which is essential for catalytic activity located in motif 9, the alanine (absent in the consensus motif but highly conserved in other PTPs) which is located at the phosphate-binding loop (residue 1394 in human HD-PTP sequence) is replaced by a serine (S) (Fig. 2A). This sequence divergence is well preserved among different HD-PTP orthologues and is likely to inhibit the enzymatic activity. Indeed, the conserved phosphate-binding loop serine residue has been suggested to play an essential role in stabilizing the enzyme-substrate complex (Fig. 2A) [24]. Furthermore, this residue is replaced by aspartic acid (D) in two known inactive receptor PTPs (PTP IA2 and PTP IA2β). It was reported that the back mutation of this residue restores PTP activity [24]–[26]. To address this possibility, back mutation of this residue (S1394A: S/A) was generated by site-directed mutagenesis, and its effect on HD-PTP catalytic activity was assessed (Fig. 2B–D). In contrast to HD-WT and CD-WT, the S1394A mutants (CD-S/A and HD-S/A) displayed significant phosphatase activity (Fig. 2C, D).

To rule out pH dependent effects on PTP activity in the wild type HD-PTP, we performed enzymatic assays in the pH range between pH 4 to 8.5 using the catalytic domain and compared this activity to the S/A mutant (Fig. 2E, F). While the CD-S/A mutant harbored enzymatic activity in the entire pH range tested with an optimal activity at pH 6.5, the CD-WT was inactive at all pH values tested demonstrating that the conserved HD-PTP sequence divergence in the phosphate-binding loop renders HD-PTP inactive. We also tested the activity of HD-PTP WT and S/A against p-nitrophenyl phosphate (*p*NPP), a synthetic substrate frequently used to evaluate PTP activity. Unfortunately, this substrate was not sensitive enough to detect HD-PTP catalytic activity (data not shown).

### Inhibition of HD-PTP S/A activity

**Figure 3:**
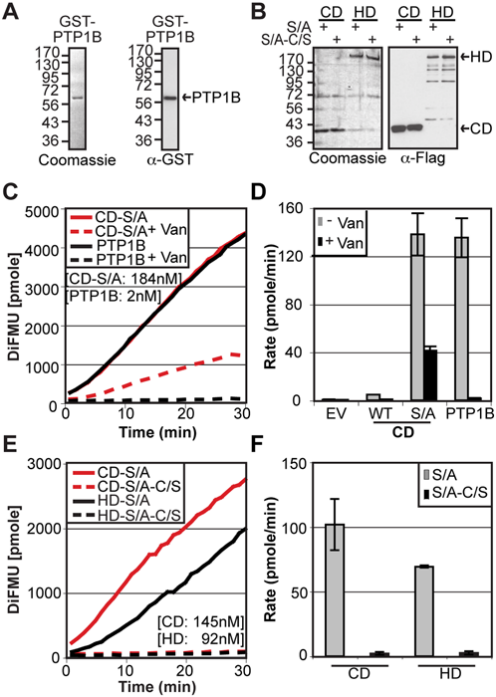
Inhibition of the HD-PTP S/A activity. A) GST-tagged catalytic domain of PTP1B, used in C and D was expressed in bacteria and purified as described [15]. The integrity and the purity of the recombinant protein were visualized by Coomassie staining and western blot analysis using anti-GST antibody. B) Flag-HD-PTP catalytic domain (CD) or full-length (HD) proteins bearing the S1394A (S/A) in combination or not with the C1392S (C/S) mutation were expressed, purified and visualized as previously described and used in E and F. C, D) The effect of the tyrosine phosphatase inhibitor sodium orthovanadate (Van) on HD-PTP catalytic domain S1394A mutant (CD-S/A) was assayed. PTP1B was used as a control of the inhibitor. E, F) The effect of a C1392S mutation on the HD-PTP catalytic domain or full-length S1392A mutant activity was assayed using DiFMUP phosphatase assay as described in figure 1. Representative kinetic curves (C, E) and average rate of reaction derived from three independent experiments (+/−SD) (D, F) are shown.

To confirm the tyrosine phosphatase specificity of the HD-PTP S/A catalytic activity, we evaluated the capacity of the well-described PTP inhibitor sodium othovanadate (Van) to inhibit the CD-S/A activity (Fig. 3 C, D). Our results show that the inhibitor efficiently abrogates the activity of the HD-PTP S/A mutant as well as the protein tyrosine phosphatase 1B isolated PTP domain (PTP1B) used as a control. In addition, we evaluated the effect of a C1392S (C/S) mutation on the HD-PTP S1394A (S/A) activity (Fig. 3 E, F). As expected, the mutation of the catalytically essential cysteine located in the PTP motif 9 abolishes PTP activity of the S/A mutants, confirming typical tyrosine phosphatase activity.

### Enzymatic parameters of HD-PTP S/A

To characterize the PTP activity of the S/A mutants, a detailed enzyme kinetic characterization was undertaken. The PTP activity of the S/A mutants (CD or HD) was determined using increasing concentrations of DiFMUP substrate (between 0 and 300 µM) and kinetic constants were derived and compared to parameters previously determined for the catalytic domain of other PTPs (Table 1). Although kinetic constants depend largely on experimental conditions, our results suggest that HD-PTP S/A affinity for substrate (*K*_M_) is similar compared to that of other PTPs (PTP1B, SHP2, TC-PTP) however, its catalytic rate (*k*_cat_) is lower resulting in a lower catalytic efficiency (*k*_cat_/*K*_M_).

**Table 1:** Kinetic constants of HD-PTP S/A and other PTPs using DiFMUP substrate.

		*K*_M_ (µM)	*k*_cat_ (s^−1^)	*k*_cat_/*K*_M_
HD-PTP	CD-S/A	18	0.1	0.3×10^4^
	HD-S/A	28	0.2	0.6×10^4^
PTP1B	Welte *et al.*	20	17	84×10^4^
	Montalibet *et al.*	5.5	28	509×10^4^
SHP2	Welte *et al.*	104	1.3	1.3×10^4^
	Montalibet *et al.*	27	12	44×10^4^
TC-PTP	Welte *et al.*	26	21	79×10^4^
	Montalibet *et al.*	11	65	591×10^4^

### Lipid phosphatase activity

**Figure 4:**
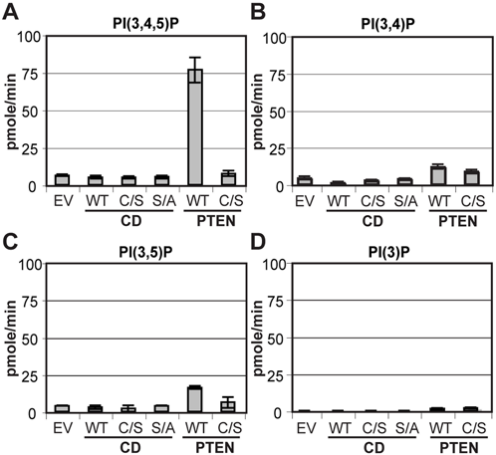
HD-PTP does not harbor lipid phosphatase activity. HD-PTP phosphatase activity toward synthetic phosphatidylinositol phosphate substrates was evaluated using a malachite green-based assay. The catalytic domain (CD) of HD-PTP wild type (WT), C1392S (C/S) or S1394A (S/A) were incubated with PI(3,4,5)P (A), PI(3,4)P (B), PI(3,5)P (C) and PI(3)P (D) and phosphate release was quantified as described in Materials and Methods. PTEN wild type (WT) and C132S (C/S) purified proteins were used as controls. Results represent the average of three independent experiments (+/−SD).

HD-PTP has been classified as a tyrosine-specific PTP based on its amino acid sequence but our results clearly demonstrated that it does not harbor any tyrosine phosphatase activity against the synthetic substrates DiFMUP and *p*NPP or a library of 38 phospho-peptides [13]. Some PTP members of the PTEN and myotubularin families have evolved to dephosphorylate phosphatidylinositol phosphates specifically [2], [27]. For example, purified PTEN specifically catalyzes dephosphorylation of PI(3,4,5)P substrates [16]. The HD-PTP sequence does not resemble a lipid phosphatase based on well-described essential residues in PTEN, however other critical residues have been identified in PTPRQ [28]. Indeed, the lipid phosphatase activity of PTPRQ appears to be dependent on a WPE sequence in its PTP motif 8 that differs from the conserved WPD consensus sequence. Since HD-PTP also possesses a WPE sequence, we assessed its ability to dephosphorylate phosphatidylinositiol phosphates using a malachite green-based assay (Fig. 4) However, our results show that unlike wild type PTEN (WT), HD-PTP is inactive towards PI(3,4,5)P substrates (Fig. 4A). The lack of HD-PTP lipid phosphatase activity also is also observed with other phosphatidylinositol phosphates including PI(3,4)P, PI(3,5)P or PI(3)P (Fig. 4B–D).

### HD-PTP effect on colony growth reduction

**Figure 5:**
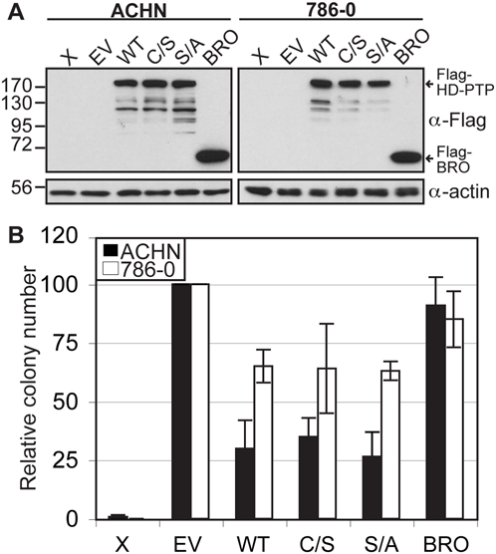
HD-PTP reduces colony growth formation independently of its phosphatase activity status. Colony formation assays were performed in renal cell carcinoma lines (ACHN and 786-0), transfected with an empty vector (EV) containing a neomycin resistance gene or expressing Flag-HD-PTP constructs as indicated. The protein expression levels were analyzed by western blot using anti-Flag antibody and anti-actin antibody as a loading control (A). B) Colonies obtained after 2 weeks of G418 selection were fixed, stained and counted. Results are expressed as the number of colonies relative to the EV condition and represent the average of independent experiments (+/−SD), (786-0: n = 4, ACHN: n = 5). X: untransfected cells.

Previous work has shown that HD-PTP expression inhibits *ras*-mediated cell transformation in NIH 3T3 cells and that deletion of the catalytic domain as well as the C/S mutation abolishes this property [4]. This result is inconsistent with the catalytic inactivity of HD-PTP described here. Using a similar approach, we addressed the effect of HD-PTP S/A mutation *in vivo* by evaluating the ability of HD-PTP to inhibit colony growth formation in human cancer cell lines. In agreement with its kidney tumor suppressor potential in humans, we recently reported that mouse HD-PTP is strongly expressed in the epithelial cells of renal tubules of the kidney cortex (Gingras *et al.* in press). We chose two renal carcinoma cell lines (ACHN and 786-0) that originated from this location in the kidney to address the tumor suppressor potential of HD-PTP. Cells were transfected with the same amount of HD-PTP constructs containing a neomycin selection gene and were selected with a neomycin analog (G418) for 2 weeks. Expression levels of all proteins were comparable using western blot analysis (Fig. 5A). The number of emerging colonies was compared with the number of colonies obtained with the empty vector (EV) control. Results show that HD-PTP expression leads to a reduction of colony growth formation in both cell lines (∼70% in ACHN and ∼40% in 786-0) (Fig. 5B). However, we did not observe any difference between the WT, the C/S and the S/A mutant suggesting that the status of the HD-PTP tyrosine phosphatase activity does not influence its negative effect on colony growth formation. Strikingly, the expression of a deletion mutant of HD-PTP, containing the BRO domain but not the HIS and PTP domains, did not affect colony growth formation. This suggests that the PTP domain exhibits functional importance as demonstrated earlier by the NIH3T3 experiments using the rat orthologue of HD-PTP [4].

## Discussion

While HD-PTP has been described as a classical non-transmembrane PTP based on its amino acid sequence, our results demonstrate that it is a catalytically inactive tyrosine phosphatase. Using a similar mammalian expression system, a recent study reported tyrosine phosphatase activity associated with wild type HD-PTP which was similar to the background activity that we detected in our assays [11]. Their results are supported by the fact that the well described PTP inhibitor sodium orthovanadate abrogated 60% of this activity. However, these results were not compared with activities of inactivating mutants (C/S), which served as controls in our assays. Strikingly, we show that the catalytically inactive C/S mutant displays the same level of PTP activity as the wild type protein. Therefore, we are confident that the low level of PTP activity detected in both studies is due to co-purification of contaminating phosphatases. In agreement with results reported here it has been shown recently that bacterially expressed HD-PTP catalytic domain was inert against a panel of 38 phosphopeptides and DiFMUP substrate [13]. Our data strongly suggest that HD-PTP is catalytically inactive due to a conserved non-consensus key amino acid divergence in its PTP motif 9, since a back mutation of this residue (S/A) reactivates the HD-PTP tyrosine phosphatase activity. The importance of this residue for the tyrosine phosphatase activity has also been reported for two other receptor-like PTPs [24], [29]. Therefore, we propose to modify the PTP motif 9 consensus by adding the alanine in position C+2 to the consensus sequence (VHCS**A**GXGR[T/S]G).

The HD-PTP sequence displays several differences from the PTP consensus motifs, therefore HD-PTP could be inactivated at multiple key positions and it would be interesting to evaluate if other reconstitutions of PTP consensus motifs could increase the HD-PTP catalytic efficiency. For example, in the PTP motif 1 (**N**XXKNR**Y**), known as a phosphotyrosine recognition loop that restricts the substrate specificity to tyrosine-phosphorylated peptide, HD-PTP (**Y**SLKNR**H**) displays divergences that are also observed in the two inactive receptor-like PTPs IA2 and IA2β [1]. Another important alteration with the PTP consensus is located in motif 8 (WP**D**XGXP), where the aspartic acid (D) is replaced by a glutamic acid (E) in the HD-PTP sequence [1]. This residue is known as the general acid catalyst and its replacement by an alanine residue considerably reduced PTP activity in PTP1B [30]. This alteration is also present in a number of inactive D2 domains in receptor PTPs. However, back mutation of this residue (E/D) was not sufficient to render HD-PTP catalytically active (data not shown).

**Figure 6:**
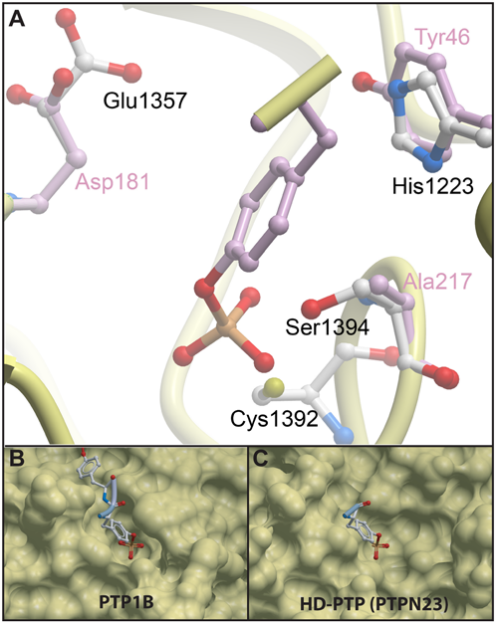
Model of the HD-PTP catalytic domain. A) The HD-PTP homology model was based on the structure of a PTP1B substrate complex (pdb-code: 1g1h). HD-PTP residues are labeled in black and PTP1B residues in grey, respectively. Shown are conserved residues known to play key roles in PTP substrate recognition and catalysis that are altered in HD-PTP. B–C) Surface representations of the PTP1B active site associated with two phosphate residues (B) and prediction of the HD-PTP protein associated with one phosphate (C).

In order to rationalize the effect of the conserved residue alterations in HD-PTP, we generated a homology model (Fig. 6A). In contrast to alanine in that position, Ser1394 in HD-PTP does not form favorable substrate interactions with the bound phosphotyrosine. The polar Ser residue may also repel the substrate phosphate group and affect the nucleophilic nature of the adjacent catalytic Cys residue. It is also evident that the tyrosine/histidine (His1223) exchange in the HD-PTP phosphotyrosine recognition loop results in the loss of π-π stacking interactions with the substrate. In addition, surface representation of the PTP1B active site associated with two phosphotyrosine residues (Fig. 6B) has been compared to a HD-PTP prediction model associated with one phosphate (Fig. 6C). The surface representation suggests that like PTP1B, HD-PTP has an open binding pocket in its active site that may allow phosphate binding.

In addition to the inactive phosphatases IA2 and IA2β, a number of other proteins possessing inactive PTP domains have been described. The second PTP domain (D2) of many classical receptor-like PTPs and the PTP domain of dual-specificity pseudophosphatases of the myotubularin family lack critical residues essential for their catalytic activity [1], [27]. However, the functional role of these inactive domains is still uncharacterized. Inactivating PTP mutants have been shown to preserve their capacity to bind substrates and have been used to trap and identify specific substrates in cells [31], [32]. The inactive catalytic domain of HD-PTP could therefore act as a phospho-tyrosine binding module, also called STYX domain, which may control cell signaling pathways by preventing the dephosphorylation of its binding partners or by regulating their cellular localization [27], [33].

In agreement with a tumor suppressor function, we observed that HD-PTP expression reduces colony growth formation independently of its PTP activity. However, expression of a HD-PTP mutant devoid of HIS and PTP domains did not affect colony formation suggesting functional importance of the PTP domain. This was also demonstrated earlier using the rat orthologue of HD-PTP in *ras* transformed NIH3T3 cells [4].

Recently, it has been shown that disruption of the fly orthologue of HD-PTP resulted in a defect in photoreceptor differentiation regulated by EGFR [4], [12]. Strikingly, the re-expression of the WT and C/S mutant rescued the defect equally well. This data further substantiates our results that HD-PTP function in cell signaling is independent of a tyrosine phosphatase activity.

## Supporting Information

Figure S1 Primers sequences: Sense primers containing an EcoR1 site (underlined) and antisense primers containing an Xho1 site (underlined) used to amplified HD-PTP full-length or catalytic domain only. Sense and antisense primers used for site-directed mutagenesis of HD-PTP full-length and catalytic domain. Nucleotide replacements are indicated in bold.
(7.62 MB TIF)

## References

[1] Andersen JN, Mortensen OH, Peters GH, Drake PG, Iversen LF (2001). Structural and evolutionary relationships among protein tyrosine phosphatase domains.. Mol Cell Biol.

[2] Alonso A, Sasin J, Bottini N, Friedberg I, Friedberg I (2004). Protein tyrosine phosphatases in the human genome.. Cell.

[3] Tabernero L, Aricescu AR, Jones EY, Szedlacsek SE (2008). Protein tyrosine phosphatases: structure-function relationships.. Febs J.

[4] Cao L, Zhang L, Ruiz-Lozano P, Yang Q, Chien KR (1998). A novel putative protein-tyrosine phosphatase contains a BRO1-like domain and suppresses Ha-ras-mediated transformation.. J Biol Chem.

[5] Hesson LB, Cooper WN, Latif F (2007). Evaluation of the 3p21.3 tumour-suppressor gene cluster.. Oncogene.

[6] Imreh S, Klein G, Zabarovsky ER (2003). Search for unknown tumor-antagonizing genes.. Genes Chromosomes Cancer.

[7] Ji L, Minna JD, Roth JA (2005). 3p21.3 tumor suppressor cluster: prospects for translational applications.. Future Oncol.

[8] Toyooka S, Ouchida M, Jitsumori Y, Tsukuda K, Sakai A (2000). HD-PTP: A novel protein tyrosine phosphatase gene on human chromosome 3p21.3.. Biochem Biophys Res Commun.

[9] Castiglioni S, Maier JA, Mariotti M (2007). The tyrosine phosphatase HD-PTP: A novel player in endothelial migration.. Biochem Biophys Res Commun.

[10] Doyotte A, Mironov A, McKenzie E, Woodman P (2008). The Bro1-related protein HD-PTP/PTPN23 is required for endosomal cargo sorting and multivesicular body morphogenesis.. Proc Natl Acad Sci U S A.

[11] Mariotti M, Castiglioni S, Garcia-Manteiga JM, Beguinot L, Maier JA (2008). HD-PTP inhibits endothelial migration through its interaction with Src.. Int J Biochem Cell Biol.

[12] Miura GI, Roignant JY, Wassef M, Treisman JE (2008). Myopic acts in the endocytic pathway to enhance signaling by the Drosophila EGF receptor.. Development.

[13] Barr AJ, Ugochukwu E, Lee WH, King ON, Filippakopoulos P (2009). Large-scale structural analysis of the classical human protein tyrosine phosphatome.. Cell.

[14] Welbourn S, Green R, Gamache I, Dandache S, Lohmann V (2005). Hepatitis C virus NS2/3 processing is required for NS3 stability and viral RNA replication.. J Biol Chem.

[15] Stuible M, Zhao L, Aubry I, Schmidt-Arras D, Bohmer FD (2007). Cellular Inhibition of Protein Tyrosine Phosphatase 1B by Uncharged Thioxothiazolidinone Derivatives.. Chembiochem.

[16] Maehama T, Dixon JE (1998). The tumor suppressor, PTEN/MMAC1, dephosphorylates the lipid second messenger, phosphatidylinositol 3,4,5-trisphosphate.. J Biol Chem.

[17] Gordon JA (1991). Use of vanadate as protein-phosphotyrosine phosphatase inhibitor.. Methods Enzymol.

[18] Peters KG, Davis MG, Howard BW, Pokross M, Rastogi V (2003). Mechanism of insulin sensitization by BMOV (bis maltolato oxo vanadium); unliganded vanadium (VO4) as the active component.. J Inorg Biochem.

[19] Welte S, Baringhaus KH, Schmider W, Muller G, Petry S (2005). 6,8-Difluoro-4-methylumbiliferyl phosphate: a fluorogenic substrate for protein tyrosine phosphatases.. Anal Biochem.

[20] Montalibet J, Skorey KI, Kennedy BP (2005). Protein tyrosine phosphatase: enzymatic assays.. Methods.

[21] Wu GS (2003). Colony growth suppression by tumor suppressor genes.. Methods Mol Biol.

[22] Peters GH, Branner S, Moller KB, Andersen JN, Moller NP (2003). Enzyme kinetic characterization of protein tyrosine phosphatases.. Biochimie.

[23] Chiarugi P, Taddei ML, Ramponi G (2005). Oxidation and tyrosine phosphorylation: synergistic or antagonistic cues in protein tyrosine phosphatase.. Cell Mol Life Sci.

[24] Jiang S, Tulloch AG, Kim TA, Fu Y, Rogers R (1998). Characterization and chromosomal localization of PTP-NP-2, a new isoform of protein tyrosine phosphatase-like receptor, expressed on synaptic boutons.. Gene.

[25] Fitzgerald LR, Walton KM, Dixon JE, Largent BL (1997). PTP NE-6: a brain-enriched receptor-type protein tyrosine phosphatase with a divergent catalytic domain.. J Neurochem.

[26] Magistrelli G, Toma S, Isacchi A (1996). Substitution of two variant residues in the protein tyrosine phosphatase-like PTP35/IA-2 sequence reconstitutes catalytic activity.. Biochem Biophys Res Commun.

[27] Tonks NK (2006). Protein tyrosine phosphatases: from genes, to function, to disease.. Nat Rev Mol Cell Biol.

[28] Oganesian A, Poot M, Daum G, Coats SA, Wright MB (2003). Protein tyrosine phosphatase RQ is a phosphatidylinositol phosphatase that can regulate cell survival and proliferation.. Proc Natl Acad Sci U S A.

[29] Drake PG, Peters GH, Andersen HS, Hendriks W, Moller NP (2003). A novel strategy for the development of selective active-site inhibitors of the protein tyrosine phosphatase-like proteins islet-cell antigen 512 (IA-2) and phogrin (IA-2beta).. Biochem J.

[30] Flint AJ, Tiganis T, Barford D, Tonks NK (1997). Development of “substrate-trapping” mutants to identify physiological substrates of protein tyrosine phosphatases.. Proc Natl Acad Sci U S A.

[31] Blanchetot C, Chagnon M, Dube N, Halle M, Tremblay ML (2005). Substrate-trapping techniques in the identification of cellular PTP targets.. Methods.

[32] Tiganis T, Bennett AM (2007). Protein tyrosine phosphatase function: the substrate perspective.. Biochem J.

[33] Wishart MJ, Dixon JE (1998). Gathering STYX: phosphatase-like form predicts functions for unique protein-interaction domains.. Trends Biochem Sci.

