# Supplementary figures and images for: HD-PTP Is a Catalytically Inactive Tyrosine Phosphatase Due to a Conserved Divergence in Its Phosphatase Domain
Source: PLoS One. 2009 Apr 2;4(4):e5105. doi: 10.1371/journal.pone.0005105 (PMC2661844; doi:10.1371/journal.pone.0005105)

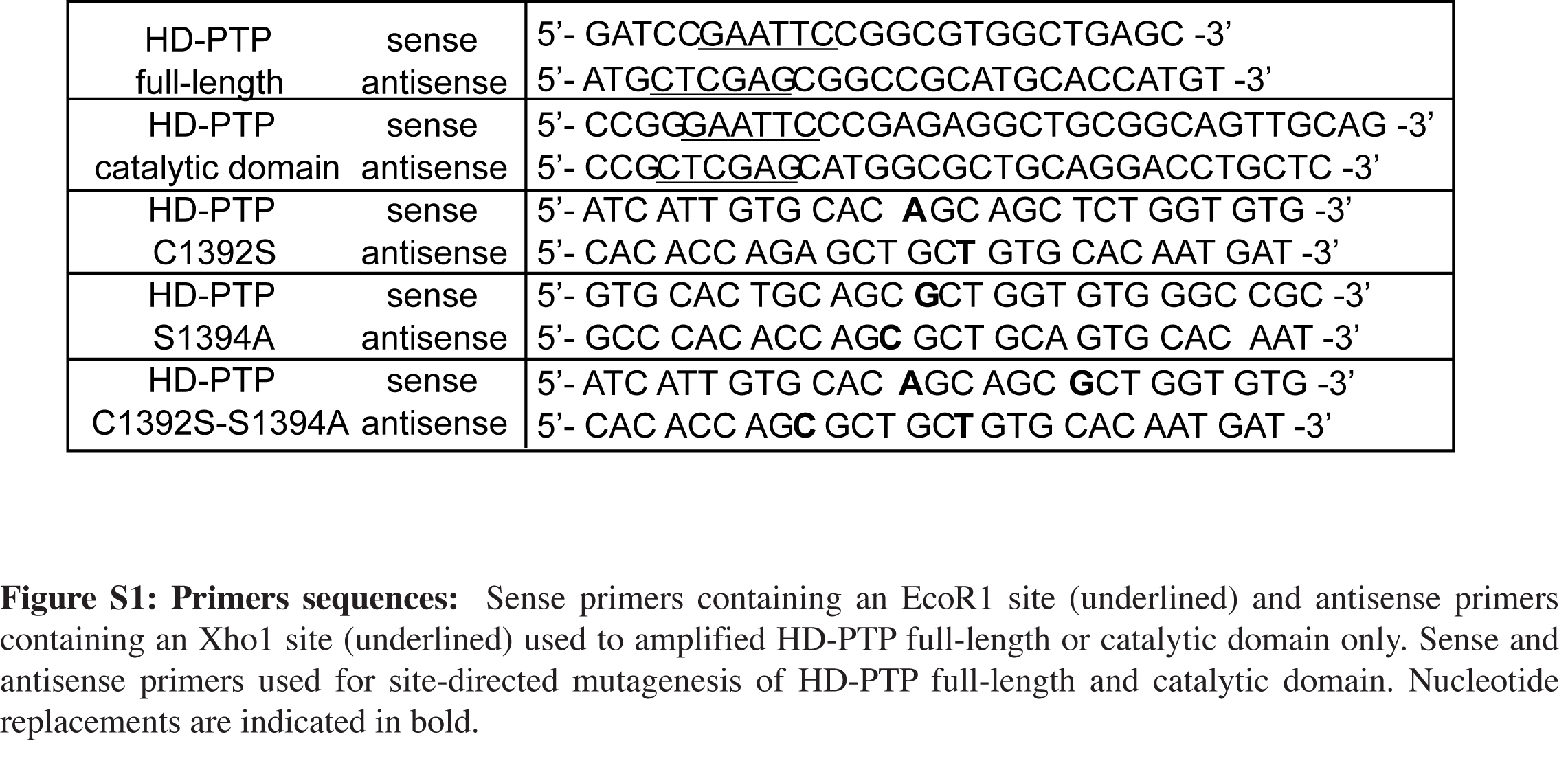

Supplement: Figure S1 — Primers sequences: Sense primers containing an EcoR1 site (underlined) and antisense primers containing an Xho1 site (underlined) used to amplified HD-PTP full-length or catalytic domain only. Sense and antisense primers used for site-directed mutagenesis of HD-PTP full-length and catalytic domain. Nucleotide replacements are indicated in bold. (7.62 MB TIF) [file pone.0005105.s001.tif]
